# Adult weight gain, fat distribution and mammographic density in Spanish pre- and post-menopausal women (DDM-Spain)

**DOI:** 10.1007/s10549-012-2108-3

**Published:** 2012-06-12

**Authors:** Marina Pollán, Virginia Lope, Josefa Miranda-García, Milagros García, Francisco Casanova, Carmen Sánchez-Contador, Carmen Santamariña, Pilar Moreo, Carmen Vidal, Mercé Peris, María Pilar Moreno, José Antonio Vázquez-Carrete, Francisca Collado, Carmen Pedraz-Pingarrón, Nieves Ascunce, Dolores Salas-Trejo, Nuria Aragonés, Beatriz Pérez-Gómez, Francisco Ruiz-Perales, DDM-Spain

**Affiliations:** 1 Cancer Epidemiology Unit, National Center for Epidemiology, Carlos III Institute of Health, Monforte de Lemos 5, 28029 Madrid, Spain; 2 Consortium for Biomedical Research in Epidemiology & Public Health (CIBER en Epidemiología y Salud Pública—CIBERESP), Carlos III Institute of Health, Madrid, Spain; 3 Valencian Breast Cancer Screening Program, General Directorate of Public Health, Valencia, Spain; 4 Public Health Research Centre (Centro Superior de Investigación en Salud Pública—CSISP), Valencia, Spain; 5 Navarre Breast Cancer Screening Program, Public Health Institute, Pamplona, Spain; 6 Castile-León Breast Cancer Screening Program, General Directorate of Public Health (Gerencia Regional de Salud—SACYL), Burgos, Spain; 7 Balearic Islands Breast Cancer Screening Program, Health Promotion for Women and Children, General Directorate of Public Health & Participation, Regional Authority for Health & Consumer Affairs, Balearic Islands, Spain; 8 Galician Breast Cancer Screening Program, Galician Regional Health Authority, A Coruña, Spain; 9 Aragon Breast Cancer Screening Program, Aragon Health Service, Zaragoza, Spain; 10 Cancer Prevention and Control Unit, Catalonian Institute of Oncology (Institut Català d’Oncologia—ICO), Barcelona, Spain

**Keywords:** Mammographic density, Adult weight gain, Fat distribution, Breast cancer

## Abstract

High mammographic density (MD) is a phenotype risk marker for breast cancer. Body mass index (BMI) is inversely associated with MD, with the breast being a fat storage site. We investigated the influence of abdominal fat distribution and adult weight gain on MD, taking age, BMI and other confounders into account. Because visceral adiposity and BMI are associated with breast cancer only after menopause, differences in pre- and post-menopausal women were also explored. We recruited 3,584 women aged 45–68 years within the Spanish breast cancer screening network. Demographic, reproductive, family and personal history data were collected by purpose-trained staff, who measured current weight, height, waist and hip circumferences under the same protocol and with the same tools. MD was assessed in the left craniocaudal view using Boyd’s Semiquantitative Scale. Association between waist-to-hip ratio, adult weight gain (difference between current weight and self-reported weight at 18 years) and MD was quantified by ordinal logistic regression, with random center-specific intercepts. Models were adjusted for age, BMI, breast size, time since menopause, parity, family history of breast cancer and hormonal replacement therapy use. Natural splines were used to describe the shape of the relationship between these two variables and MD. Waist-to-hip ratio was inversely associated with MD, and the effect was more pronounced in pre-menopausal (OR = 0.53 per 0.1 units; 95 % CI = 0.42–0.66*)* than in post-menopausal women (OR = 0.73; 95 % CI = 0.65–0.82) (*P* of heterogeneity = 0.010). In contrast, adult weight gain displayed a positive association with MD, which was similar in both groups (OR = 1.17 per 6 kg; 95 % CI = 1.11–1.23). Women who had gained more than 24 kg displayed higher MD (OR = 2.05; 95 % CI = 1.53–2.73). MD was also evaluated using Wolfe’s and Tabár’s classifications, with similar results being obtained. Once BMI, fat distribution and other confounders were considered, our results showed a clear dose–response gradient between the number of kg gained during adulthood and the proportion of dense tissue in the breast.

## Introduction

Breast cancer screening programs using mammography are well extended in most developed countries, though there are variations in periodicity and age groups targeted. Mammograms reveal the characteristics of breast composition, because stroma and epithelium attenuate X-rays more than does fat and so appear light, whereas fat appears dark [1].

The term mammographic density refers to the proportion of radiologically dense breast tissue, composed of stroma and epithelium, and is a marker of susceptibility to breast cancer [2, 3]. Even though breast density is a highly heritable trait [4], it is also influenced by well-established breast cancer risk factors, such as menarche, parity, benign breast disease and hormonal replacement therapy (HRT) with estrogen and progestin [2, 5, 6]. However, mammographic density decreases with age, reflecting a reduction in the amount of stromal and epithelial tissues in the breast [7, 8]. Mammographic density also decreases with BMI, as the greater fat content associated with higher BMI reduces the proportion of dense tissue in the mammographic image [9].

BMI is a well-established risk factor for post-menopausal breast cancer, even though it is inversely correlated with incidence of breast cancer among pre-menopausal women [10–12]. Furthermore, epidemiologic studies support the idea of abdominal fatness and adult weight gain as contributing causes of post-menopausal breast cancer, even after BMI is taken into account [10, 13]. Several studies have reported an inverse correlation between abdominal fatness and mammographic density in pre- and post-menopausal women [14–21]. However, relatively few studies have examined the association between adult weight gain and mammographic density [19, 20]. Samimi et al. [19] observed an inverse correlation between pounds gained since age 18 years and percentage of dense tissue, in both pre- and post-menopausal participants in the Nurses’ Health Study, even after adjusting for BMI. A similar association was reported in US Chinese women but disappeared when BMI was taken into account [20]. This second study also showed a strong positive correlation between weight gain and amount of dense tissue, which remained statistically significant after adjusting for BMI and other anthropometric variables [20].

In this study, we analyze the influence of adult weight gain and fat distribution on mammographic density in Spanish pre- and post-menopausal women attending breast cancer screening.

## Materials and methods

### Study population

The DDM-Spain study (*Determinantes de la Densidad Mamográfica en España*—Determinants of Mammographic Density in Spain) is a cross-sectional multicenter study based on 3,584 women, aged 45–68 years, recruited from seven specific screening centers within the Spanish Breast Cancer Screening Program network in the following Spanish Autonomous Regions: Aragon; Balearic Isles; Castile-Leon; Catalonia; Galicia; Navarre; and Valencia. Women were recruited from October 7, 2007 through July 14, 2008. All women aged 50–69 years, regardless of nationality or legal status, are screened under these government-sponsored programs every 2 years. In some regions, women aged between 45 and 49 years are also included. Women were contacted by telephone and invited to participate in the study. Those who agreed to be recruited were given an appointment with the interviewer at the screening center on the same day as that scheduled for their mammogram. Participants signed an informed consent. More details regarding the design of the study are provided elsewhere [5, 22].

Women were interviewed at the screening center by purpose-trained interviewers. The questionnaire collected demographic data, family and personal background information, including weight at age 18, and gynecologic, obstetric and occupational history. A food frequency questionnaire referring to the preceding year was administered. Bra size was also ascertained. An anthropometric examination of the participants was conducted following standardized procedures. Women’s waist, hip, height and weight were measured twice by the interviewer, with a third measurement being taken if the first two were not similar. Identical types and models of balance scale, stadiometer and measuring tape were used at all the study centers. Waist circumference was measured at the midpoint between the lowest rib and the iliac crest, and hip circumference was measured around the widest portion of the buttocks. Average anthropometric values were used in the analysis. Body mass index (BMI) was estimated as weight in kg divided by the square of height in meters.

Menopausal status was self-reported and based on four specific questions: (1) What is your situation as regard menstruation? (answers were, “I have regular menstruations,” “I have started to have irregularities,” or “I am post-menopausal”); (2) How many periods have you had in the last 12 months? (3) Age at menopause (this was only addressed to women who considered themselves to be post-menopausal); and (4) cause of menopause. Post-menopausal status was then defined as absence of menstruation in the last 12 months. Women were re-interviewed by telephone and inconsistencies between questions were resolved. The few participants who had their uterus, but not their ovaries, surgically removed were classified as post-menopausal, if they were aged older than 49 years, because this is the average age of menopause in Spain.

Four of the screening centers used analog mammography devices, while the other three used full-field digital machines. Mammographic density was assessed from the craniocaudal mammogram of the left breast using a visual semiquantitative score with six categories proposed by Boyd [23], namely, A (0 %), B (<10 %), C (10–25 %), D (25–50 %), E (50–75 %) and F (>75 %). All mammograms were read by a single experienced radiologist in a blinded manner. To test the reliability of our radiologist, a subsample of the mammograms was assessed a second time, showing a high concordance between the first and second readings (weighted kappa value of 0.92) [24]. The same radiologist read the whole set of mammograms using two other qualitative classifications frequently used in the literature, i.e., the Wolfe and Tabár Scales [25, 26]. Every mammogram was read randomly in each case, to prevent recall bias, until every scale was completed. The information of previous readings was not available [24]. Wolfe’s and Tabár’s classifications were only used to confirm the results obtained in the final model.

### Statistical methods

The association between MD, adult weight gain and fat distribution was evaluated by using ordinal logistic models with random center-specific intercepts [27]. Ordinal logistic regression, also known as the proportional-odds model, assumes that odds ratios (ORs) remain constant, irrespective of the cut-off chosen to dichotomize the ordinal classification of MD into two groups, i.e., high versus low MD. The model simultaneously estimates as many equations as the number of categories in the dependent variable minus one. The main explanatory variables of interest were (1) adult weight gain, defined as the number of kg of difference between weight reported at age 18 and current measured weight and (2) fat distribution, considering waist and hip circumferences, and waist-to-hip and waist-to-height ratios. All models were initially adjusted for age, BMI, bra size, parity, time since menopause, family history of breast cancer (only first-degree relatives were considered) and use of hormonal replacement therapy (current, past or none). The Brant test was used to verify this proportional-odds assumption [28]. The random term accounted for unexplained heterogeneity associated with the screening center, including differences between interviewers and mammographic devices.

The correlation between adult weight gain, BMI and the remaining variables related with fat distribution was computed using Pearson’s correlation coefficients. Waist-to-hip ratio was selected as the overall measurement of fat distribution, given that it was less closely correlated with BMI than were the rest. The final model included adult weight gain, waist-to-hip ratio together with the rest of the abovementioned variables, except for use of hormonal replacement therapy because this exposure was not associated with MD in our study. For the purpose of constructing the final model, to be able to compute adult weight gain for women who failed to remember their weight at age 18 years (15 %), this variable was imputed as the median weight at age 18 reported by women drawn from the same screening center, height quintile and age group. Similarly, for the few women who did not know their bra size (0.7 %), this was imputed by taking the median size reported by women drawn from the same screening center and BMI quintile. A sensitivity analysis was performed but imputed information was excluded, to verify that such imputation did not alter the estimated effect of adult weight gain and waist-to-hip ratio on mammographic density. To test the consistency of the effect of these two anthropometric variables across categories of BMI, the final model was separately fitted for women stratified by observed BMI quintile.

The final model was also separately fitted for pre- and post-menopausal women. Heterogeneity of effects between pre- and post-menopausal women was tested, by using the log-likelihood ratio test to compare the final model with a model that also included an interaction term between menopausal status and the corresponding explanatory variable. Furthermore, natural splines were used to explore the shape of the dose–response curve for the two variables of interest, waist-to-hip ratio and adult weight gain, without assuming a linear dose–response relationship. Splines were constructed using 4 knots, located in Harrell’s recommended percentiles, namely, 5, 35, 65 and 95 % [29]. These spline models included all the explanatory variables considered in the final model and were fitted separately for pre- and post-menopausal women.

To explore the consistency of the results yielded by using different density scales, the same model, including adult weight gain, waist-to-hip ratio, age, BMI, parity, family history of breast cancer and time since menopause, was fitted using Wolfe’s and Tabár’s classifications of mammographic density. Separate analyses were also performed on the pre- and post-menopausal groups, and heterogeneity of effects between these two groups was likewise checked.

All analyses were performed in Stata (StataCorp L.P, College Station, TX), using the *glamm* function to fit random-intercept ordinal logistic models [30].

## Results

A total of 3,584 women were recruited and interviewed, with an average participation rate of 74.5 % (range 64.7–84.0 %). Ten women developed breast cancer within 6 months of mammography and were excluded from the analysis. Table 1 shows the characteristics of the participants according to their menopausal status. The average ages of pre- and post-menopausal women were 49 and 58 years, respectively. Post-menopausal participants were less frequently nulliparous (8.6 vs. 10.4 %) and registered higher parity figures, with 31 % reporting more than three deliveries compared with only 18 % among pre-menopausal women. Seven percentage of participants reported having at least one first-degree relative with breast cancer, with the proportion being similar in both groups. Only 3 % of post-menopausal women were taking hormonal therapy at the date of mammographic screening, while another 10 % had used this type of treatment previously. Pre-menopausal women were taller (mean height of 158 vs. 156 cm) and less obese (BMI of 27 vs. 28), showing statistically significant differences in all measurements of fat distribution compared to the post-menopausal group. While both groups reported an average weight of 53 kg at age 18 years, weight gain thereafter proved to be greater in the post-menopausal group (16 kg vs. 14 kg). More than 40 % of pre-menopausal, as compared with only 18 % of post-menopausal women, had an MD greater than 50 %. MD assessment was not available for a total of 16 women, 5 pre-menopausal and 11 post-menopausal; all these women were eliminated from the subsequent analyses.

**Table 1:** Socio-demographic characteristics and anthropometric measurements among DDM participants by menopausal status

	Post-menopausal (*N* = 2,754)	Pre-menopausal (*N* = 820)	*P* value
Age (mean, SD)	58 (4.5)	49 (2.9)	<0.001
Parity, *N* (%)			<0.001
Nuliparous	237 (8.6)	85 (10.4)	
One	371 (13.5)	174 (21.2)	
Two	1298 (47.1)	418 (50.9)	
Three	603 (21.9)	114 (13.9)	
Four or more	245 (8.9)	29 (3.5)	
First-degree relative with breast cancer, *N* (%)	202 (7.3)	58 (7.1)	0.800
Hormonal replacement therapy, *N* (%)			<0.001
No	2402 (87.2)	812 (99.0)	
Current use	82 (3.0)	7 (0.9)	
Past use	270 (9.8)	1 (0.1)	
Years since menopause, *N* (%)			
<5 years	748 (27.2)		
5–10	867 (31.5)		
10–15	663 (24.1)		
>15	476 (17.3)		
Bra size, *N* (%)			<0.001
80–85	297 (10.8)	121 (14.8)	
90	480 (17.4)	178 (21.7)	
95	786 (28.5)	229 (27.9)	
100	508 (18.5)	151(18.4)	
105	353 (12.8)	75 (9.2)	
110	200 (7.3)	44 (5.4)	
115–120	115 (4.2)	20 (2.4)	
Unknown	15 (0.5)	2 (0.2)	
Anthropometric variables			
BMI (mean, SD)	28.3 (5.0)	27.0 (4.9)	<0.001
Waist (mean, SD)	88.6 (11.6)	84.8 (11.7)	<0.001
Hip (mean, SD)	105.2 (9.6)	103.6 (9.6)	<0.001
Height (mean, SD)	156.3 (5.9)	158.2 (5.7)	<0.001
Waist-to-hip ratio (mean, SD)	0.84 (0.07)	0.82 (0.07)	<0.001
Waist-to-height ratio (mean, SD)	0.57 (0.08)	0.54 (0.08)	<0.001
Weight at age 18 (mean, SD)	52.7 (7.9)	52.9 (7.2)	0.529
Current Weight (mean, SD)	69.2 (12.3)	67.5 (12.4)	0.001
Weight gain (kg) since age 18 (mean, SD)	16.1 (12.2)	14.1 (11.0)	<0.001
Mammographic density, *N* (%)			<0.001
A: 0 %	141 (5.1)	10 (1.2)	
B: <10 %	626 (22.7)	98 (12.0)	
C: 10–25 %	625 (22.7)	108 (13.2)	
D: 25–50 %	870 (31.6)	269 (32.8)	
E: 50–75 %	375 (13.6)	249 (30.4)	
F: >75 %	106 (3.9)	81 (9.9)	
Not available	11 (0.4)	5 (0.6)	

Table 2 shows the distribution of MD extreme categories and ORs obtained for anthropometric measurements, adjusted for age, BMI, time since menopause, parity, family history of breast cancer, use of hormonal replacement therapy and bra size. The linear trend for each of these characteristics is also provided, with the categorical variable being replaced by a continuous variable. All fat distribution–related measures showed a strong inverse association with MD, except for hip circumference, which failed to show any association. Weight at age 18 years was also inversely associated with MD. A total of 552 women were unable to report their weight at that age and they are included in the table as a separate category. Interestingly, adult weight gain showed a positive effect on MD, e.g., compared to women with a weight difference of less than 6 kg, those who had gained 24 kg or more had an OR of 1.86 (95 % CI 1.40–2.44). The last part of the table shows the ORs for the adjustment variables. As expected, MD decreased with age, time since menopause, number of deliveries and bra size. There was a positive association between family history of breast cancer and MD (OR: 1.29; *P* value = 0.033), but no differences in MD were observed between women who reported current or past use of hormonal menopausal therapy and non-users.

**Table 2:** Association between anthropometric variables and other characteristics of the study population and mammographic density (Boyd’s semiquantitative classification)

		Mammographic density				
Variable	*N*	<10 % (A + B) (%)	>50 % (E + F) (%)	OR^ab^	95 % CI^ab^	*P* value^ab^
Anthropometric variables						
BMI (kg/m^2^)						
<23.9	712	9	43	1.00		
23.9–26.3	711	15	31	0.71	0.58–0.86	<0.001
26.3–28.5	711	22	21	0.52	0.42–0.64	<0.001
28.5–31.7	711	32	13	0.35	0.28–0.43	<0.001
>31.7	711	46	6	0.22	0.17–0.28	<0.001
*Per 1*				*0.88*	*0.87*–*0.89*	<*0.001*
Waist (cm)						
<77.8	711	8	44	1.00		
77.8–84.0	749	16	30	0.85	0.68–1.06	0.142
54.1–90.0	667	21	19	0.65	0.51–0.83	0.001
90.1–97.1	710	33	12	0.52	0.39–0.70	<0.001
>97.1	709	46	8	0.49	0.35–0.68	<0.001
*Per 10*				*0.66*	*0.59*–*0.74*	<*0.001*
Hip (cm)						
< 97	719	12	40	1.00		
97.1–101.6	700	17	27	0.94	0.76–1.16	0.575
101.7–105.9	711	22	23	1.02	0.81–1.28	0.888
106.0–112.0	731	28	15	1.00	0.77–1.30	0.980
> 112.0	682	45	9	1.07	0.77–1.47	0.691
*Per 10*				*1.00*	*0.93*–*1.07*	*0.948*
Waist-to-hip ratio						
< 0.78	709	9	40	1.00		
0.78–0.82	709	20	27	0.79	0.65–0.96	0.018
0.82–0.85	707	22	22	0.73	0.60–0.89	0.002
0.85–0.89	709	33	13	0.53	0.43–0.65	<0.001
>0.89	709	38	11	0.54	0.43–0.67	<0.001
*Per 0.1 increase*				*0.69*	*0.62*–*0.77*	<*0.001*
Waist-to-height ratio						
< 0.49	711	7	47	1.00		
0.49–0.53	707	14	30	0.72	0.57–0.90	0.004
0.53–0.57	708	23	18	0.52	0.40–0.67	<0.001
0.57–0.62	710	30	13	0.44	0.33–0.59	<0.001
> 0.62	710	48	7	0.34	0.24–0.48	<0.001
*Per 0.1 increase*				*0.49*	*0.41*–*0.59*	<*0.001*
Weight at age 18 (kg)						
< 48	675	21	28	1.00		
48–50	759	19	29	1.04	0.86–1.26	0.693
51–54	451	22	22	0.82	0.66–1.02	0.069
55–59	578	23	21	0.84	0.69–1.04	0.108
> 59	544	36	13	0.58	0.47–0.72	0.000
Unknown	549	29	20	0.89	0.72–1.10	0.263
*Per 5* *kg increase*				*0.89*	*0.85*–*0.93*	<*0.001*
Adult weight gain (kg)						
<6	576	15	34	1.00		
6–12	631	15	29	1.24	1.01–1.52	0.044
12–18	664	22	24	1.34	1.08–1.66	0.007
18–24	500	29	20	1.44	1.13–1.83	0.003
>24	636	38	11	1.86	1.40–2.44	0.000
Unknown	549	29	20	1.43	1.13–1.80	0.003
*Per 6* *kg of gain*				*1.18*	*1.11*–*1.24*	<*0.001*
Other variables						
Age (years)						
<50	548	10	42	1.00		
50–54	973	20	28	0.75	0.61–0.92	0.006
55–59	1,001	25	18	0.6	0.51–0.79	<0.001
60–64	939	37	12	0.50	0.39–0.65	<0.001
≥65	95	38	15	0.52	0.33–0.82	0.004
*Per 5* *years*				*0.85*	*0.78*–*0.93*	<*0.001*
Time since menopause (years)						
0	819	13	41	1.00		
<5	739	21	23	0.68	0.560.83	<0.001
5–10	863	27	17	0.57	0.46–0.71	<0.001
10–15	659	33	15	0.53	0.41–0.69	<0.001
>15	476	35	13	0.49	0.36–0.66	<0.001
*Per 5* *years*				*0.89*	*0.83*–*0.95*	<*0.001*
Parity						
Nuliparous	319	15	35	1.00		
1	542	18	33	0.76	0.59–0.98	0.038
2	1,708	24	22	0.56	0.445–0.71	<0.001
3	716	29	18	0.53	0.41–0.67	<0.001
4	188	36	6	0.37	0.26–0.51	<0.001
5 or more	83	41	4	0.26	0.14–0.41	<0.001
*Per birth*				*0.80*	*0.75*–*0.84*	<*0.001*
Family history of breast cancer						
No	3,297	25	22	1.00		
Yes	260	21	29	1.29	1.02–1.63	0.033
Hormonal Replacement Therapy						
No	3,200	24	23	1.00		
Current use	88	23	24	0.75	0.50–1.11	0.145
Past use	270	31	18	0.86	0.68–1.08	0.189
Bra size						
80–85	418	9	43	1.49	1.20–1.85	<0.001
90	654	16	30	1.04	0.87–1.24	0.695
95	1,011	22	25	1.00		
100	656	28	15	0.90	0.75–1.08	0.262
105	424	35	13	0.85	0.69–1.06	0.151
110	242	41	8	0.87	0.66–1.14	0.310
115–120	134	56	4	0.62	0.43–0.89	0.009
Unknown	17	29	24	0.83	0.34–2.02	0.679
*Bra size per 5* *cm*				*0.90*	*0.86*–*0.94*	<*0.001*

^a^ORs and 95 % confidence interval (95 % CI) adjusted for age, BMI, bra size, time since menopause, parity, family history of breast cancer and hormonal replacement treatment use

^b^In *italics* ORs 95 %CI and *P* values obtained with the corresponding variable as a continuous term

**Fig. 1:**
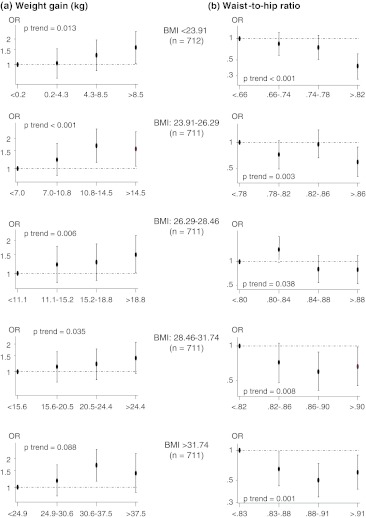
The effect of adult weight gain and waist-to-hip ratio on MD, estimated separately for groups of women defined by observed BMI quintile

All anthropometric variables were strongly and statistically significantly correlated with BMI, with Pearson’s correlation coefficients equal to or >0.80 for all of these, except bra size (0.63) and waist-to-hip ratio (0.43). To avoid a multi-colinearity problem, we decided to use waist-to-hip ratio as an indicator of fat distribution in subsequent analyses, because it was the measure showing the least correlation with BMI. Figure 1 graphically shows the effect of adult weight gain and waist-to-hip ratio on MD, separately estimated for groups of women defined by observed BMI quintile. For each subgroup of women, the corresponding quartiles of the two variables, weight gain and waist-to-hip ratio, were considered in these analyses. Imputed values for weight at age 18 years and bra size were used to prevent the exclusion of a substantial number of women (564) from the analysis. ORs were adjusted for all the abovementioned variables except hormonal replacement therapy. In all BMI categories, a positive dose–response gradient was observed for adult weight gain in relation to MD, even though the trend was not statistically significant in more obese women. For fat distribution, the inverse association between MD and waist-to-hip ratio was confirmed in all BMI groups.

**Fig. 2:**
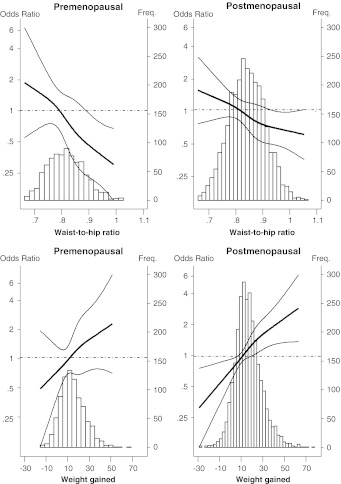
The dose–response curve for the main variables of interest, waist-to-hip ratio and adult weight gain, in pre- and post-menopausal women, respectively, using natural splines

The joint analysis of adult weight gain, fat distribution and the remaining explanatory variables is shown in Table 3, both overall and stratified by menopausal status. In this final model, age, BMI, bra size and parity were included together as continuous variables. Both anthropometric variables (adult weight gain and waist-to-hip ratio) were included in this model, and were thus adjusted for each other. The last column of the table refers to the statistical significance of the interaction term between menopausal status and the pertinent explanatory factor introduced in the model as a continuous variable. These results are supplemented by Fig. 2, which separately depicts the dose–response curve for the main variables of interest, waist-to-hip ratio and adult weight gain, in pre- and post-menopausal women, respectively, using natural splines. These graphs also include the histogram with the distribution of women, shown on the same scale for both groups. Although waist-to-hip ratio was inversely associated with MD, the effect was more pronounced among pre-menopausal women (ORs among pre- and post-menopausal of 0.53 and 0.73, respectively, for an increase of 0.1), with the interaction term being statistically significant (*P* value = 0.010). Indeed, the downward trend seemed to be attenuated among post-menopausal women with a higher waist-to-hip ratio (Table 4; Fig. 2). In contrast, adult weight gain displayed a positive association with MD, which was similar in both pre- and post-menopausal women (OR of 1.17 per 6 kg. of weight gain). Women who gained more than 24 kg. had an OR of 2.05 (95 % CI = 1.53-2.73). The remaining variables likewise showed effects that were similar in pre- and post-menopausal women. The sensitivity analysis performed after removing the imputed data yielded results which were very similar to those shown in Table 3 for the two variables of interest, i.e., waist-to-hip ratio (OR = 0.72 for an increase of 0.1; 95 % CI = 0.64-0.81) and adult weight gain (OR = 1.19 for an increase of 6 kg; 95 % CI = 1.12–1.26). The interaction term between menopausal status and waist-to-hip ratio remained statistically significant (*P* value = 0.005), with the effect of this variable being more pronounced in pre-menopausal (OR = 0.55 for an increase of 0.1; 95 % CI = 0.43–0.70) than in post-menopausal women (OR = 0.78 for an increase of 0.1; 95 % CI = 0.69–0.89).

**Table 3:** ORs, 95 % confidence intervals and P values for higher mammographic density (Boyd’s semiquantitative classification) associated with waist-to-hip ratio, adult weight gain and other characteristics of the study population, by menopausal status

Variable	All women			Pre-menopausal women			Post-menopausal women			Heterogeneity^c^
	OR^ab^	95 % CI^ab^	*P* value^ab^	OR^ab^	95 % CI^ab^	*P* value^ab^	OR^ab^	95 % CI^ab^	*P* value^ab^	
Waist-to-hip ratio										
<0.78	1.00			1.00			1.00			
0.78–0.82	0.75	0.62–0.91	0.004	0.85	0.59–1.22	0.377	0.73	0.58–0.92	0.007	
0.82–0.85	0.70	0.57–0.85	<0.001	0.59	0.40–0.86	0.006	0.75	0.59–0.95	0.015	
0.85–0.89	0.49	0.40–0.60	<0.001	0.37	0.24–0.57	<0.001	0.53	0.42–0.67	<0.001	
>0.89	0.51	0.41–0.63	<0.001	0.29	0.18–0.47	<0.001	0.57	0.45–0.73	<0.001	
*Trend per 0.1 units*	*0.68*	*0.61*–*0.76*	<*0.001*	*0.53*	*0.42*–*0.66*	<*0.001*	*0.73*	*0.65*–*0.82*	<*0.001*	*0.010*
Weight gained (kg)										
<6	1.00			1.00			1.00			
6–12	1.29	1.06–1.57	0.012	1.11	0.75–1.63	0.613	1.36	1.08–1.72	0.009	
12–18	1.51	1.23–1.86	<0.001	1.34	0.87–2.04	0.178	1.58	1.24–2.01	<0.001	
18–24	1.63	1.28–2.07	<0.001	1.74	1.05–2.89	0.033	1.65	1.26–2.16	<0.001	
>24	2.05	1.53–2.73	<0.001	1.43	0.77–2.35	0.253	2.31	1.66–3.21	<0.001	
*Trend per 6* *kg of gain*	*1.17*	*1.11*–*1.23*	<*0.001*	*1.16*	*1.03*–*1.31*	*0.014*	*1.17*	*1.10*–*1.24*	<*0.001*	*0.223*
BMI										
Per 1 kg/m^2^	0.86	0.84–0.88	<0.001	0.85	0.81–0.89	<0.001	0.86	0.84–0.88	<0.001	*0.157*
Bra size										
Per 5 cm	0.93	0.88–0.97	0.002	1.02	0.91–1.14	0.709	0.90	0.85–0.95	<0.001	0.766
Age										
Per 1 year	0.97	0.96–0.99	<0.001	0.92	0.88–0.97	0.001	0.89	0.80–0.99	0.023	0.577
Number of births										
Per 1 birth	0.81	0.76–0.85	<0.001	0.89	0.78–1.02	0.091	0.78	0.73–0.83	<0.001	0.141
Time since menopause										
Pre-menopausal	1.00			–	–	–	–			
<5 years	0.67	0.55–0.82	<0.001	–	–	–	1.00			
5–10 years	0.56	0.45–0.70	<0.001	–	–	–	0.82	0.68–0.99	0.046	
10–15 years	0.53	0.41–0.69	<0.001	–	–	–	0.77	0.61–0.96	0.019	
>15 years	0.48	0.36–0.64	<0.001	–	–	–	0.69	0.54–0.89	0.004	
*Trend per 5* *years*	*0.88*	*0.83*–*0.94*	<*0.001*	–	–	–	*0.91*	*0.84*–*0.98*	*0.011*	
Family history of breast cancer										
No	1.00			1.00			1.00			
Yes	1.32	1.04–1.67	0.020	1.40	0.85–2.31	0.091	1.30	1.00–1.70	0.050	0.945

^a^ORs and 95 % confidence interval (95 % CI) from the multivariate model including all variables presented in the table

^b^In *italics* ORs 95 % CI and *P* values obtained with the corresponding variable as a continuous term

^c^ *P* value of the interaction term between menopausal status and the corresponding variable introduced in the model as a continuous term

**Table 4:** ORs, 95 % confidence intervals and *P* values for waist-to-hip ratio and adult weight gain associated with higher mammographic density, using Wolfe’s and Tabar’s classifications, by menopausal status

Variable	All women			Pre-menopausal women			Post-menopausal women			Heterogeneity^c^
Wolfe classification^a^, *n* (%)										
NI		314 (9)			39 (5)			275 (10)		
P1		1514 (43)			227 (28)			1287 (47)		
P2		1135 (32)			295 (36)			840 (31)		
DY		594 (17)			254 (31)			340 (12)		

Variable	OR^bc^	95 % CI^bc^	*P* value^bc^	OR^dc^	95 % CI^dc^	*P* value^dc^	OR^bc^	95 % CI^bc^	*P* value^bc^	Heterogeneity^e^
Waist-to-hip ratio										
<0.78	1.00			1.00			1.00			
0.78–0.82	0.69	0.56–0.84	<0.001	0.74	0.51–1.07	0.117	0.67	0.53–0.85	0.001	
0.82–0.85	0.70	0.57–0.85	0.001	0.64	0.43–0.95	0.026	0.73	0.57–0.93	0.010	
0.85–0.89	0.45	0.37–0.57	<0.001	0.41	0.26–0.62	<0.001	0.48	0.37–0.61	<0.001	
>0.89	0.49	0.39–0.64	<0.001	0.33	0.20–0.54	<0.001	0.54	0.42–0.70	<0.001	
*Trend per 0.1 units*	*0.67*	*0.60*–*0.74*	<*0.001*	*0.55*	*0.44*–*0.69*	<*0.001*	*0.71*	*0.63*–*0.80*	<*0.001*	*0.031*
Weight gained (kg)										
<6	1.00			1.00			1.00			
6–12	1.14	0.93–1.40	0.204	0.96	0.64–1.44	0.848	1.20	0.94–1.53	0.147	
12–18	1.33	1.07–1.65	0.009	1.06	0.68–1.63	0.796	1.47	1.15–1.89	0.002	
18–24	1.44	1.13–1.85	0.004	1.35	0.80–2.27	0.256	1.55	1.60–2.06	0.003	
>24	1.86	1.37–2.52	<0.001	1.14	0.61–2.14	0.687	2.23	1.57–3.15	<0.001	
*Trend per 6* *kg of gain*	*1.14*	*1.08*–*1.21*	<*0.001*	*1.09*	*0.96*–*1.23*	*0.170*	*1.16*	*1.09*–*1.24*	<*0.001*	*0.164*
Tábar classification^f^, *n* (%)										
II		313 (9)			39 (5)			274 (10)		
III		1842 (52)			298 (37)			1544 (56)		
IV		1146 (32)			386 (47)			760 (28)		
V		256 (7)			92 (11)			164 (6)		

Variable	OR^bc^	95 % CI^bc^	*P* value^bc^	OR^dc^	95 % CI^dc^	*P* value^dc^	OR^bc^	95 % CI^bc^	*P* value^bc^	Heterogeneity^e^
Waist-to-hip ratio										
<0.78	1.00			1.00			1.00			
0.78–0.82	0.68	0.56–0.84	<0.001	0.72	0.48–1.07	0.102	0.66	0.52–0.85	0.001	
0.82–0.85	0.72	0.58–0.89	0.003	0.62	0.41–0.94	0.026	0.75	0.59–0.98	0.028	
0.85–0.89	0.49	0.39–0.62	<0 .001	0.47	0.30–0.74	0.001	0.49	0.38–0.64	<0.001	
>0.89	0.54	0.43–0.68	<0.001	0.37	0.22–0.62	<0.001	0.58	0.45–0.76	<0.001	
*Trend per 0.1 units*	*0.71*	*0.64*–*0.80*	<*0.001*	*0.61*	*0.48*–*0.77*	<*0.001*	*0.74*	*0.65*–*0.85*	*0.001*	*0.425*
Weight gained (kg)										
<6	1.00			1.00	1.00		1.00			
6–12	1.14	0.92–1.41	0.216	0.97	0.64–1.47	0.892	1.20	0.93–1.54	0.150	
12–18	1.37	1.09–1.71	0.006	1.22	0.77–1.92	0.397	1.43	1.10–1.85	0.007	
18–24	1.41	1.09–1.82	0.009	1.37	0.80–2.35	0.254	1.45	1.08–1.96	0.014	
>24	1.49	1.09–2.05	0.013	0.99	0.51–1.92	0.985	1.70	1.18–2.44	0.004	
*Trend per 6* *kg of gain*	*1.11*	*1.05*–*1.18*	<*0.001*	*1.06*	*0.94*–*1.20*	*0.359*	*1.13*	*1.06*–*1.21*	<*0.001*	*0.541*

^a^Wolfe classification

N1: Breast composed almost completely of fat, with perhaps just a few fibrous connective tissue strands

P1: Breast composed mainly of fat, although up to a quarter of the sub-areolar area may show beaded or cord-like areas of ducts

P2: More severe involvement of the breast, with a prominent duct pattern occupying more than one quarter of breast volume

DY: Breast typically contains extensive regions of homogeneous mammographic densities. The proportion of density is greater than that of the fat

^b^ORs and 95 % confidence interval (95 % CI) adjusted for age, BMI, time since menopause, parity and family history of breast cancer

^c^In *italics* ORs 95 %CI and *P* values obtained with the corresponding variable as a continuous term

^d^ORs and 95 % confidence interval (95 % CI) adjusted for age, BMI, and family history of breast cancer

^e^ *P* value of the interaction term between menopausal status and the corresponding variable introduced in the model as a continuous term

^f^Tabár classification

I: Mammogram composed of scalloped contours with some lucent areas of fatty replacement and 1-mm evenly distributed nodular densities (none of our mammograms were classified in this category)

II: Mammogram composed almost entirely of lucent areas of fatty replacement and 1-mm evenly distributed nodular densities

III: Prominent ducts in the retroareolar area

IV: Extensive, nodular and linear densities with nodular size larger than normal lobules

V: Homogeneous ground-glass-like appearance with no perceptible features

Table 4 lists the ORs, 95 % confidence intervals and *P* values for waist-to-hip ratio and adult weight gain, on fitting the final models shown in Table 4 but considering the two qualitative density classifications proposed by Wolfe and Tabár, respectively. The results were very similar when the Wolfe scale was used, and the interaction between waist-to-hip ratio and menopausal status remained statistically significant (*P* value = 0.031). The association between these variables and density classified according to Tabár’s scale was less pronounced, however, and the dose–response trend for weight gain among pre-menopausal women was no longer in evidence.

## Discussion

Our results show that, after adjusting for BMI, bra size and other possible confounders, adult weight gain was positively correlated with mammographic density, in both pre- and post-menopausal women. A strong inverse association was also observed between abdominal fat distribution, as measured by waist-to-hip ratio, and mammographic density.

Previous studies have already reported an inverse association between abdominal fat distribution and mammographic density [14–21]. A recent study using whole-body dual X-ray absorptiometry and axial computed tomography to estimate fat mass and abdominal adipose tissue has confirmed that the inverse association between adiposity and percentage of MD was explained by a strong positive correlation with the nondense area/volume, together with an inverse and weaker correlation with the dense area/volume [9]. Anthropometric measures of adiposity, such as waist circumference, and those taken with imaging methods were similarly associated with the mammographic measures [9]. A weak inverse correlation between adiposity and dense area has been observed by some [14, 17, 21] but not all studies [16, 20]. The association between adiposity and nondense area is expected because the breast is one of women’s bodies’ fat depots. Bra size was also inversely associated with breast density and positively correlated with BMI, adult weight gain and fat distribution. We included this variable in the model in an attempt to allow for the increased volume of the breast associated with fat storage. Bra cup information was requested but 37 % of our subjects were not able to answer this question, probably because of the difficulty of finding different bra cups in Spanish lingerie stores until quite recently.

An interesting result of our study is the different effect of visceral adiposity on mammographic density in pre- versus post-menopausal women, with a steeper inverse correlation observed in the former group. The higher percentage of mammographic density observed in our study among women with a lower waist-to-hip ratio, for any BMI quintile, and the steeper effect in pre-menopausal women may be a marker of higher accumulated exposure to estrogens. Sex steroid hormones play important roles in the accumulation, metabolism and distribution of adipose tissue, creating a sexual dimorphic pattern [31–33]. Female fat distribution is signaled by the waist-to-hip ratio; estrogens stimulate the accumulation of fat in the gluteal and femoral areas, creating the characteristic gynoid phenotype [31, 34]. By early adulthood, sex differences in body shape are maximal [31]. As adulthood progresses, sex steroid levels decline because of an increase in sex hormone binding proteins, which reduces the concentration of the free form [31]. The process of aging is associated with substantial redistribution of fat tissue among depots [35]. Visceral fat varies inversely with estrogen levels [36], and the cessation of gonadal estrogen production at menopause is associated with the emergence of a more android pattern [31]. Treatment with estrogen in post-menopausal women, however, restores the lipoprotein lipase activity of the femoral but not the abdominal adipocytes [37], and post-menopausal women who were receiving HRT had a lower waist circumferences [38]. Thus, the gynoid fat phenotype among pre-menopausal women might be a surrogate of a greater cumulative estrogenic exposure, determined by genetic mechanisms and/or reproductive factors, such as early menarche, low parity and others that are associated with both mammographic density and breast cancer risk.

On the other hand, the strong inverse correlation between mammographic density and visceral adiposity partially explains the lack of connection between breast cancer incidence and BMI or fat distribution in pre-menopausal women. Adjustment for mammographic density has, in fact, been shown to reverse the direction of this association [39, 40]. Among post-menopausal women, however, both obesity and visceral adiposity increase the risk of breast cancer [10, 11]. After menopause, ovarian estrogen biosynthesis is replaced by peripheral site synthesis, and the visceral adipose tissue is the main source of estrogens [41]. Several years after menopause, waist circumference is positively correlated with blood estrogen levels [42]. Obesity and mammographic density seem to operate through separate pathways [39]. A panel of experts recommended the inclusion of these two factors in breast cancer prediction models [43].

With regard to adult weight gain, our results indicated a positive association between this variable and mammographic density, once age, BMI and the remaining confounders had been taken into account. Previous studies have reported an inverse correlation between weight gain and mammographic density [19]. A recent study targeting US Chinese women reported a positive association between adult weight gain and the dense area in the mammogram solely among women with a BMI of less than 23 [20]. In contrast, a 2-year intervention study using a low-fat high-carbohydrate diet observed that, while there was a decrease in the mammographic dense area associated with weight loss, the percentage of density actually increased [44]. Whereas all these studies used computer-assisted quantitative methods to determine mammographic density, we used a single experienced radiologist with high intra-observer concordance [24]. The same results were observed on using qualitative measures of mammographic density, such as the Wolfe and Tabár scales, which take the mammographic pattern into account. Nevertheless, the precise extent to which different methods of assessing mammographic density might explain the above differences between our and others’ results is difficult to judge. Computer-assisted methods delimit the breast area using the line of the skin, whereas our radiologist focused on the mammary gland and tended to disregard subcutaneous fat.

Adult weight gain is thought to be a better measure than BMI when it comes to assessing adiposity and its metabolic consequences, because weight gain largely reflects an increase in body fat independent of BMI [45]. A recent meta-analysis has confirmed the association between adult weight gain and post-menopausal breast cancer, particularly for ER+ PR+ tumors [13]. Studies with transgenic mice containing the human aromatase gene have shown that weight gain stimulates local aromatase expression in the breast increasing the local amount of estrogens [46]. This phenomenon is accentuated in ovariectomized mice [47]. Furthermore, weight loss through caloric restriction or gastric bypass surgery reduces the concentration of circulating estrogens in post-menopausal women [41]. This mechanism may link adult weight gain with an increase in mammographic density, because it has been shown that estrogens are the major epithelial cell mitogen in adult non-pregnant women [48]. Adult weight gain accounts for >20 % of all post-menopausal invasive breast tumors in the US [49]. This is also an important modifiable risk factor in our context: Spanish women in our study gained an average of 400 g per year since the age of 18 years, and one in five women currently weighed 24 kg or more than when she was 18 years old.

Age, parity and time elapsed since menopause significantly decreased mammographic density. Family history of breast cancer was positively associated with mammographic density: It was considered a potential confounder, owing to the reported association between anthropometric variables, particularly higher waist circumference, and family history [50]. Finally, hormonal replacement therapy was not associated with mammographic density in our study. It should be noted that only 3 % of the post-menopausal women in our study were on hormonal therapy at the date of mammographic examination, and most of these (71 %) used estrogen-only treatment.

The DDM-Spain is the largest epidemiologic study analyzing mammographic density and breast cancer risk factors in Spanish women. Women were recruited from population-based Spanish breast cancer screening centers, and participation rates were high. According to the data supplied by the Spanish National Health Survey [51], our women were very similar to the national sample in the same age range, in terms of life-style factors, such as smoking habits, alcohol consumption and use of hormonal replacement therapy. Prevalence of obesity was higher in our study (29.5 vs. 24.9 %), but information in the National Health Survey is based on self-reported data, which implies a substantial underestimation of BMI [52]. The ordinal nature of the dependent variable was taken into account, by using ordinal logistic regression rather than traditional logistic models, which entail a loss of valuable information by combining different density categories.

Our study has also a series of limitations. First, one of our variables, adult weight gain, critically depended on subjects’ ability to remember their weight at age 18 years, something that 15 % of our women were unable to recall. Computing adult weight gain in such women using imputed weights at age 18 years implies a certain degree of non-differential misclassification. Indeed, as expected, the exclusion of imputed values resulted in steeper trends. Second, anthropometric measures were obtained by the pertinent interviewer following the standard protocol. The use of different interviewers at the recruiting screening centers introduces a certain amount of heterogeneity. The random effects term sought to take this unmeasured variability into account. Third, the number of pre-menopausal women might have been insufficient for the purpose of detecting significant differences in some associations, such as the dose–response curve for adult weight gain. This constraint was imposed by the type of women screened in Spain. It should be borne in mind that European breast-screening guidelines recommend mammographic examination in the age range from 50 to 69 years, and only three of our screening centers included women in their 40s. Finally, measurement of density was performed visually by a single radiologist using categorical scales. The use of quantitative methods has been recommended [43]. Such methods are not free of subjectivity, however, and, while they are validated for analog mammograms, their performance with digital mammograms is not well established. In our study, three of the participant centers used digital images. Quantitative methods afford the chance to study the association between obesity-related factors and the absolute area of dense and non-dense breast tissue, which may in turn shed light on mechanistic pathways. Information obtained by visual classification is related only with the relative amount and specific pattern of the dense tissue. Nevertheless, density percentages and patterns are the established risk markers of increased breast cancer risk [2], and our results were highly consistent, whether based on a semi-quantitative method, such as Boyd’s scale, or alternatively, on qualitative classifications widely used in the literature, such as Wolfe’s and Tabár’s scales.

Our results confirm an inverse association between fat distribution and mammographic density, which was more pronounced in pre-menopausal women. Once BMI and fat distribution were taken into account, however, adult weight gain was positively associated with mammographic density in our subjects. This positive association appeared using both quantitative and qualitative density methods, and reflects a state of higher breast cancer susceptibility.

## Appendix

### Other members of DDM-Spain

Gonzalo López-Abente, Pablo Fernández-Navarro Anna Cabanes (National Center for Epidemiology, Instituto de Salud Carlos III, Madrid, Spain; Consortium for Biomedical Research in Epidemiology & Public Health CIBERESP, Spain), Soledad Laso, Manuela Alcaraz (Valencia Breast Cancer Screening Program, General Directorate Public Health, Valencia, Spain; Centro Superior de Investigación en Salud Pública CSIP, Valencia, Spain), María Ederra (Navarra Breast Cancer Screening Program, Public Health Institute, Pamplona, Spain; Consortium for Biomedical Research in Epidemiology & Public Health CIBERESP, Spain), Isabel González-Román (Health and Social Welfare Department, Section for Health Promotion and Protection, Castile-León, Spain), Ana-Belén Fernández, Montserrat Corujo (Galicia Breast Cancer Screening Program, Regional Authority of Health, Galicia Regional Government, Santiago, Spain), Soledad Abad (Aragón Breast Cancer Screening Program, Health Service of Aragón, Zaragoza, Spain), and Jesús Vioque (Universidad Miguel Hernandez, Alicante, Spain; Consortium for Biomedical Research in Epidemiology & Public Health CIBERESP, Spain).

## Abbreviations

BMI: Body mass index
DDM-Spain: Determinants of Mammographic Density in Spain
MD: Mammographic density
HRT: Hormonal replacement treatment
OR: Odds ratio
95 % CI: 95 % confidence interval
